# Exploring the impact of adolescent cognitive inflexibility on emotional and behavioural problems experienced by autistic adults

**DOI:** 10.1177/13623613211046160

**Published:** 2021-09-28

**Authors:** Matthew J Hollocks, Tony Charman, Gillian Baird, Catherine Lord, Andrew Pickles, Emily Simonoff

**Affiliations:** 1 King’s College London, UK; 2 South London and Maudsley NHS Foundation Trust, UK; 3 Evelina Children’s Hospital, Guy’s and St Thomas’ NHS Foundation Trust, UK; 4 UCLA Semel Institute for Neuroscience and Human Behavior, USA; 5 King’s College London and Biomedical Research Centre for Mental Health, UK

**Keywords:** anxiety, autism spectrum disorders, cognition (attention and learning, memory), depression, psychiatric comorbidity

## Abstract

Autistic young people experience high levels of co-occurring mental health difficulties, including anxiety, depression and behavioural difficulties, across their lifespan. Understanding the neuropsychological mechanisms which underlie these difficulties is vital in developing personalised supports and interventions. Cognitive inflexibility is one candidate mechanism which is associated with co-occurring mental health comorbidities but is also associated with other features of autism such as restricted and repetitive behaviours. This study investigates the distinct longitudinal association between cognitive inflexibility, measured using objective neuropsychological measures and emotional and behavioural problems across adolescence and early adulthood. Eighty-one autistic people from a population-based longitudinal study were assessed at 16 and 23 years on measures of emotional and behavioural problems, with cognitive inflexibility, restricted and repetitive behaviours and verbal intelligence quotient measured at 16 years. We used structural equation modelling to investigate the relationship between cognitive inflexibility and emotional and behavioural symptoms at both timepoints while accounting for the severity of restricted and repetitive behaviours and verbal intelligence quotient. Our results indicate an effect of cognitive inflexibility on increased behavioural problems at 16 years and emotional problems across timepoints, which is distinct from restricted and repetitive behaviours and verbal intelligence quotient. Exploratory mediation analyses suggest that cognitive inflexibility may be one mechanism through which emotional difficulties are maintained longitudinally.

**Lay abstract:**

Autistic people experience high levels of co-occurring mental health difficulties. To develop more effective treatments, a greater understanding of the thinking processes that may lead to these difficulties is needed. Cognitive inflexibility, defined as a rigid pattern of thoughts and subsequently behaviours, is one possible thinking trait which has previously been associated with both co-occurring mental health difficulties but also other features of autism such as restricted and repetitive behaviours. Restricted and repetitive behaviours include repetitive movements, ritualistic behaviours, and/or highly focused interests. This study investigates the relationship between, cognitive inflexibility, measured using neuropsychological tasks, and emotional and behavioural problems across adolescence and early adulthood. Eighty-one autistic people who were recruited to be representative of the wider autism population were assessed at 16 and 23 years on measures of emotional and behavioural problems, with cognitive inflexibility, restricted and repetitive behaviours and verbal intelligence measured at 16 years. We used statistical modelling to investigate the relationship between cognitive inflexibility and emotional and behavioural symptoms at both timepoints while accounting for the possible relationship with restricted and repetitive behaviours and verbal intelligence quotient. Our results suggest that cognitive inflexibility may be an important factor associated with emotional difficulties across adolescence and early adulthood. This suggests that developing intervention approaches targeting cognitive inflexibility may be an important step in improving the mental health of those with autism.

## Introduction

Autism spectrum disorder (ASD) is a neurodevelopmental condition characterised by difficulties with reciprocal social communication, restricted interests and repetitive behaviours (American Psychiatric Association, 2013). In addition to these core features, it is recognised that a high proportion of autistic people have co-occurring difficulties with emotions (anxiety and low mood) and can display angry, irritable or aggressive behaviours (hereafter referred to as behavioural difficulties) for a variety of reasons including heightened anxiety, emotion regulation difficulties and environmental factors. This is in the context of several possible strengths for some autistic young people in areas such as socialisation, communication and independent living skills (Szatmari et al., 2021).

In childhood and adolescence, anxiety disorders are particularly prominent with a prevalence of around 40%, with estimates of depression being much lower (Chandler et al., 2016; Simonoff et al., 2008). However, evidence indicates that the prevalence of depression increases through adolescence (Gotham et al., 2015; McCauley et al., 2020) and into adulthood so that around 27% of adults with autism will have a diagnosis of depression or at least moderate levels of depressive symptoms (Hollocks et al., 2019). While at a population-level symptoms of emotional and behavioural difficulties as a whole seem to reduce in autistic people between childhood and early adulthood (Stringer et al., 2020), this is not true for all, and the high prevalence of co-occurring emotional and behavioural symptoms remains relatively stable throughout childhood (Simonoff et al., 2013) and into early adulthood (Woodman et al., 2016). It is unclear whether emotional and behavioural difficulties have common or overlapping aetiologies, but the high rates of co-occurrence suggest that there may be one or more underlying processes associated with a more general difficulty in emotional and behavioural regulation (Conner et al., 2020).

Autistic people show greater cognitive inflexibility when compared to those without an autism diagnosis (Strang et al., 2017), and it may be one process underlying co-occurring emotional and behavioural difficulties. At a neuropsychological level, cognitive flexibility involves efficiently disengaging from one task or mode of response, switching to another, while inhibiting the initial task response or perseverative response patterns. At a psychological level, cognitive inflexibility can be defined as the tendency to focus on one’s own thoughts, beliefs or behaviours often to the exclusion of most others (Murray et al., 2005), thus limiting flexible problem-solving or inhibition of the current thought and thus being unable to easily transition from one thought/behaviour to another (Dajani & Uddin, 2015; Ozonoff et al., 1994). Cognitive flexibility is itself considered a domain of executive functioning. Difficulties with executive functioning are common in autism (Demetriou et al., 2018) and have been shown to be related to having greater symptoms of both emotional (Hollocks et al., 2014) and behavioural difficulties (Carter Leno et al., 2019) in childhood and adolescence.

In the context of autism, cognitive inflexibility is often associated with the domain of restricted and repetitive behaviours (RRBs). Studies of autistic people, who have measured components of executive functioning associated with cognitive inflexibility (i.e. set-shifting) using neuropsychological measures, have shown significant associations with an increased severity of RRBs (South et al., 2007; Yerys et al., 2009). The precise nature of overlap between cognitive inflexibility and RRBs is unknown, but it is possible that RRBs may be the behavioural expression of an inflexible cognitive style. Furthermore, studies have identified significant associations between co-occurring anxiety symptoms and increased RRBs (Joyce et al., 2017; Rodgers et al., 2012). Therefore, it is important to understand whether the effects of cognitive inflexibility on mental health can be separated from those of RRBs and other characteristics associated with autism that have also been associated with mental health outcomes. For example, Stringer et al. (2020) found, in their analysis using this data set, that a lower intelligence quotient (IQ) and poorer language ability predicted less decline in conduct symptoms. The relationship between verbal ability and mental health difficulties more widely in autism remains unclear; with one study indicating a significant association between higher verbal IQ and more anxiety symptoms, but not depression (Gotham et al., 2015). Evidence from non-autistic children indicates that increased cognitive flexibility is also associated with both a better verbal ability and that the use of verbal strategies more broadly can enhance performance on neuropsychological measures (Cragg & Nation, 2010; Fatzer & Roebers, 2012; Kray et al., 2008). This highlights the importance of accounting for verbal ability when investigating the role of cognitive inflexibility in mental health comorbidities.

Despite the importance of cognitive inflexibility in the study of autism, there has been relatively little research directly measuring this construct and its associations with symptoms of mental health comorbidities over time. One recent study using a behavioural rating (i.e. parent-completed questionnaire) of cognitive inflexibility has shown that greater inflexibility is significantly associated with both behavioural and emotional difficulties in a sample of autistic children and adolescents (Ozsivadjian et al., 2021). A direct relationship between cognitive inflexibility and a greater frequency of behavioural difficulties was found, while the relationship with increased emotional symptoms was indirect, with less flexible young people having greater intolerance of uncertainty (Boulter et al., 2014), which significantly predicted more emotional difficulties. Intolerance of uncertainty, defined as the negative perception or interpretation of uncertain situations, is another mechanism, (Maisel et al., 2016; Pickard et al., 2020), along with difficulties with alexithymia (Milosavljevic et al., 2016) and emotional regulation (Mazefsky & White, 2014) that have been shown to be associated with emotional difficulties in autism. The implication that cognitive inflexibility is an important factor in the expression of the emotional and behavioural difficulties experienced by autistic people is supported by a limited literature showing similar associations in children, adolescents, and young adults both with (Visser et al., 2015) and without (Lawson et al., 2015; Wallace et al., 2016) mild intellectual disability.

The identification of specific cognitive processes which may relate to the aetiology, and/or severity, of both emotional and behavioural difficulties in autism is vital to continue to improve and develop interventions for this population. An example of this is intolerance of uncertainty for which specific intervention approaches have begun to be developed (Rodgers et al., 2019). Similarly, cognitive inflexibility and executive functions more generally are a promising target for intervention, as there is preliminary evidence that treatments targeting these features of autism can be used with positive results. Kenworthy et al. (2014) conducted a randomised controlled trial of a school-based intervention targeting executive functioning compared with a social-skills intervention and showed improvements in flexibility across both behavioural and cognitive measures.

However, to date, the evidence linking cognitive inflexibility with mental health symptoms is reliant on informant-reported behavioural rating questionnaires. Only one study has used an informant report and a neuropsychological measure, in this case, the Wisconsin Card Sort Task (WCST; Visser et al. (2015)), finding a significant association with behavioural problems only when using behavioural ratings of cognitive inflexibility. While behavioural rating measures can be helpful, they can also introduce bias into studies. For instance, there is the possibility of a negativity bias in which parents of the people with the most severe emotional difficulties or challenging behaviours may tend to endorse more items across a whole range of questionnaires, thus inflating the correlations between them. This poses a challenge when trying to understand the associations between highly inter-related constructs, such as cognitive inflexibility, RRBs and mental health. Objective measures such as neuropsychological assessments, which are not vulnerable to this form of bias, can be used to understand the specific role of cognitive inflexibility in expression of emotional and behavioural symptoms in autism.

This study aims to investigate whether neuropsychological measures of cognitive flexibility predict the longitudinal expression of emotional and behavioural difficulties in autism across adolescence and early adulthood. Based on previous neuropsychological and behavioural studies in childhood and adolescence, we would expect there to be a significant association cross-sectionally between inflexibility and both emotional and behavioural symptoms. The relationship with inflexibility and the expression of mental health difficulties over time is less studied, but one could hypothesise that less-flexible young people are at risk of continuing behavioural difficulties due the possible impact of inflexibility on adaptive behaviour (Bertollo et al., 2020). Using a structural equation modelling (SEM) framework, we aim to tease apart the independent effects of cognitive inflexibility on emotional and behavioural difficulties from the impact of RRBs and verbal IQ.

## Methods

### Participants

This study included 81 participants recruited as a part of the larger special needs and autism project (SNAP). SNAP includes data from 158 autistic young people and their parents, who have been followed up from childhood and into early adulthood (see Table 1 for descriptive statistics). The overall study consists of three waves of data collection at the average age of 12, 16 and 23 years. This analysis included only participants who participated at Wave 2 and who went on to be followed up at Wave 3 (see Simonoff et al., 2020 for full participant characteristics). The original cohort was derived from 56,946 children born between 1 July 1990 and 31 December 1991, in 12 districts of the South Thames region of England, United Kingdom. The sample was obtained by screening all children on the special needs register of child health services and those with a clinical ASD diagnoses using the Social Communication Questionnaire (Rutter et al., 2003) (see (Baird et al., 2006) for full details). ASD diagnoses were confirmed according to the International Classification of Diseases, Tenth Revision (ICD-10) criteria based on a full assessment, including the Autism Diagnostic Interview – Revised (Lord et al., 1994), the Autism Diagnostic Observation Schedule – Generic (Lord et al., 2000) and a detailed cognitive assessment including measures of intellectual and adaptive functioning. Specific data on socioeconomic status and culture/ethnicity were not recorded at Waves 2 and 3. The original study was reviewed and approved by the South East London Research Ethics Committee (05/MRE01/67), wave 2 by the South-East London Research Ethics Committee (05/MRE01/67) and wave 3 by the Camberwell and St. Giles NRES Committee number 12/LO/1770, IRAS project number 112286. Community stakeholders were engaged and contributed to the interpretation of key study findings.

**Table 1.** Descriptive statistics.

Variable	Mean	*SD*	Range	Data available (*n* of 81)
Age wave 2 (years)	15.4	0.45	14.7–16.8	81
Age wave 3 (years)	23.2	0.79	21.3–25.1	81
Sex (female:male)	7:74	–	–	81
Emotional/behavioural symptoms Wave 2				
SDQ emotional problems (16 years)	3.4	2.4	0–9	77
SDQ conduct problems (16 years)	2.6	2.5	1–8	77
SRS-RRBs (16 years)	32.5	13.5	7–63	76
Emotional/behavioural symptoms Wave 3				
Beck Anxiety Inventory (23 years)	6.4	7.6	0–35	79
Beck Depression Inventory (23 years)	5.6	7.4	0–32	77
SDQ conduct problems (23 years)	2.0	1.8	0–8	81
Neuropsychological measures (all Wave 2)				
Full-scale IQ	83.5	17.8	50–119	81
Verbal IQ	79.5	17.4	55–120	81
Performance IQ	81.4	21.4	45–135	81
Opposite worlds	6.16	3.67	1–14	69
Trail making (s)	65.6	46.9	13–257	72
Block design	44.7	13.3	20–68	81
Card sort (pass:fail)	59:16			81

*SD*: standard deviation; SDQ: Strengths and Difficulties Questionnaire; SRS-RRBs: Social Responsiveness Scale – Restricted and Repetitive Behaviours; IQ: intelligence quotient.

### Measures

#### Measures of mental health symptoms

##### Strengths and Difficulties Questionnaire

The Strengths and Difficulties Questionnaire (SDQ; Goodman, 1997) is an emotional and behavioural screening questionnaire consisting of 25 questions, measuring five domains: (1) emotional symptoms, (2) conduct problems, (3) hyperactivity/inattention, (4) peer relationship problems, and (5) prosocial behaviour. This analysis focuses on parent-report, which was collected at 16 and 23 years and includes only the emotional and conduct problems (as a measure of behaviour problems) sub-scales. Higher scores on the SDQ indicate more symptoms of the co-occurring mental health difficulty. The emotional symptoms sub-scale is used at 16 years only as measure of baseline emotional difficulties. The conduct problem sub-scale is used a measure of behaviour problems at 16 and 23 years.

##### Beck Anxiety Inventory

Beck Anxiety Inventory (BAI; Beck & Steer, 1990) consists of 21 items that measure the cognitive and psychological symptoms of anxiety. For this study, parent-report of their child’s anxiety symptoms was collected at 23 years. The total anxiety score was used as our anxiety outcome measure with a higher score indicating greater anxiety symptoms.

##### Beck Depression Inventory

Beck Depression Inventory (BDI; Beck & Steer, 1993) consists of 21 items that measure the severity of depressive symptoms. For this study, parent-report of their child’s depression symptoms was collected at 23 years. The total depression score was used as our depression outcome measure with a higher score indicating greater depression severity.

#### Measure of RRBs

The Social Responsiveness Scale (SRS; Constantino & Gruber, 2005) is a validated 65-item rating scale eliciting autistic behaviour over the previous 6 months. The SRS child version was used at age 16 years, which consists of total score and five sub-scales measuring different features of autism. For this study, we used the empirically derived RRB factors which include items on instance of sameness and autistic mannerisms (Frazier et al., 2014). Higher scores represent more symptoms.

#### Neuropsychological measures

The Wechsler Abbreviated Scale of Intelligence (WASI; Wechsler, 2005) was selected as a brief but reliable measure of intelligence; it contains four sub-tests that measure both verbal and non-verbal intelligence as well as providing an estimate of full-scale intelligence quotient (FSIQ). For this study, the WASI block design sub-test, a timed visual-performance task of novel problem-solving, was also used as an additional measure of cognitive flexibility, as this has previously been found to be sensitive to interventions targeting executive functions and cognitive flexibility (Kenworthy et al., 2014). We also used the estimate of verbal IQ provided in our analyses as a measure of overall verbal ability.

##### Opposite worlds

The Opposite worlds task was taken from the Test of Everyday Attention for Children (TEA-Ch; Manly et al., 1999) and was included as a measure of attentional control/flexibility and interference inhibition. The task included a ‘Same World’ trial, where the participant reads out a series of the numbers 1 and 2, and the ‘Opposite World’ trial where the participant had to say the opposite to the number they were reading (so ‘2’ when then read a 1 and ‘1’ when they read a 2). Two Same World trials and two Opposite World trials were presented. The time taken to complete each world was recorded in seconds. The outcome variable was the subtraction of the mean Same Worlds completion time from the mean Opposite Worlds completion time, with a higher score relating to cognitive inflexibility and inhibition performance.

##### Trail making

This was included as a measure of attentional switching and comprised three separate trials (Reitan, 1958). For Part A1, the participant was asked to ‘join the dots’ in numerical order 1–25. For Part A2, the participant was asked to ‘join the dots’ in alphabetic order of 25 circles labelled A–Y. For Part B1, the participant was asked to ‘join the dots’ by switching between 25 numbers and letters (i.e. 1–A–2–B–3–C and so on). The time taken to complete each trial was recorded in seconds. The outcome variable was the subtraction of Part A1 from Part B1, with a higher score indicating worse performance.

##### Card sorting task

A card-sorting task adapted from the WCST (Grant & Berg, 1948) was included as a measure of cognitive set-shifting. This version of the task was based on a previously adapted child friendly version of the WCST (Hughes et al., 1998) and full details have been published previously (Tregay et al., 2009). The task included three trials in which participants were shown a photograph of a character and a pack of 64 cards. The cards depicted single objects that varied on three dimensions: colour (e.g. red or blue), shape (e.g. squares and hearts) and size (i.e. small or large objects). Each trial required the participants to sort by a different dimension, requiring the participants to shift cognitive set. Scores ranged from 0 to 3 depending on the number of correct trials. As participants tended to either pass or fail this task, scores were re-coded ‘0’ for fail (scores of 0–2) or ‘1’ for pass (scores of 3).

### Statistical analysis

All variables were checked for normality via visual inspection using boxplots and histograms. The trail-making task and both BAI and BDI had a slight positive skew and were log transformed prior to inclusion in any analyses. Inter-correlations between key variables included in the primary analysis were examined using Pearson’s correlations and are presented in Table 2.

**Table 2.** Correlation matrix showing association between all key continuous variables.

	SDQ-E (16 years)	SDQ-C (16 years)	SDQ-C (23 years)	BAI	BDI	Trails	OW	BD	RRBs	Verbal IQ
SDQ-E (16 years)	–									
SDQ-C (16 years)	.28*	–								
SDQ-C (23 years)	.24*	.34**	–							
BAI (23 years)	.43**	.26*	.36**	–						
BDI (23 years)	.25*	.26*	.36**	.60**	–					
Trails (16 years)	.23*	.18	.41**	.23*	.29*	–				
OW (16 years)	–.21^†^	–.16	–.31**	–.37**	–.23^†^	–.49**	–			
BD (16 years)	–.29**	–.19^†^	–.32**	–.45**	–.25*	–.46**	.52**	–		
RRBs (16 years)	.38**	–.18	.14	.37**	.22^†^	.45**	–.20	–.35**	–	
Verbal IQ (16 years)	–0.02	–0.03	–0.22*	–0.16*	–0.09	–.40**	.38**	.55**	–.41**	–

SDQ-E: Strength and Difficulties Emotional problems; SDQ-C: Strength and Difficulties Conduct problems; BAI: Beck Anxiety Inventory; BDI: Beck Depression Inventory; Trails: trail-making Task; OW: Opposite Worlds Task; BD: Block Design Task; RRBs: Social Responsiveness Scale – mannerisms sub-scale; IQ: intelligence quotient.

† *p* *⩽* 0.05; *p *⩽* 0.05; **p *⩽* 0.01.

**Figure 1.**
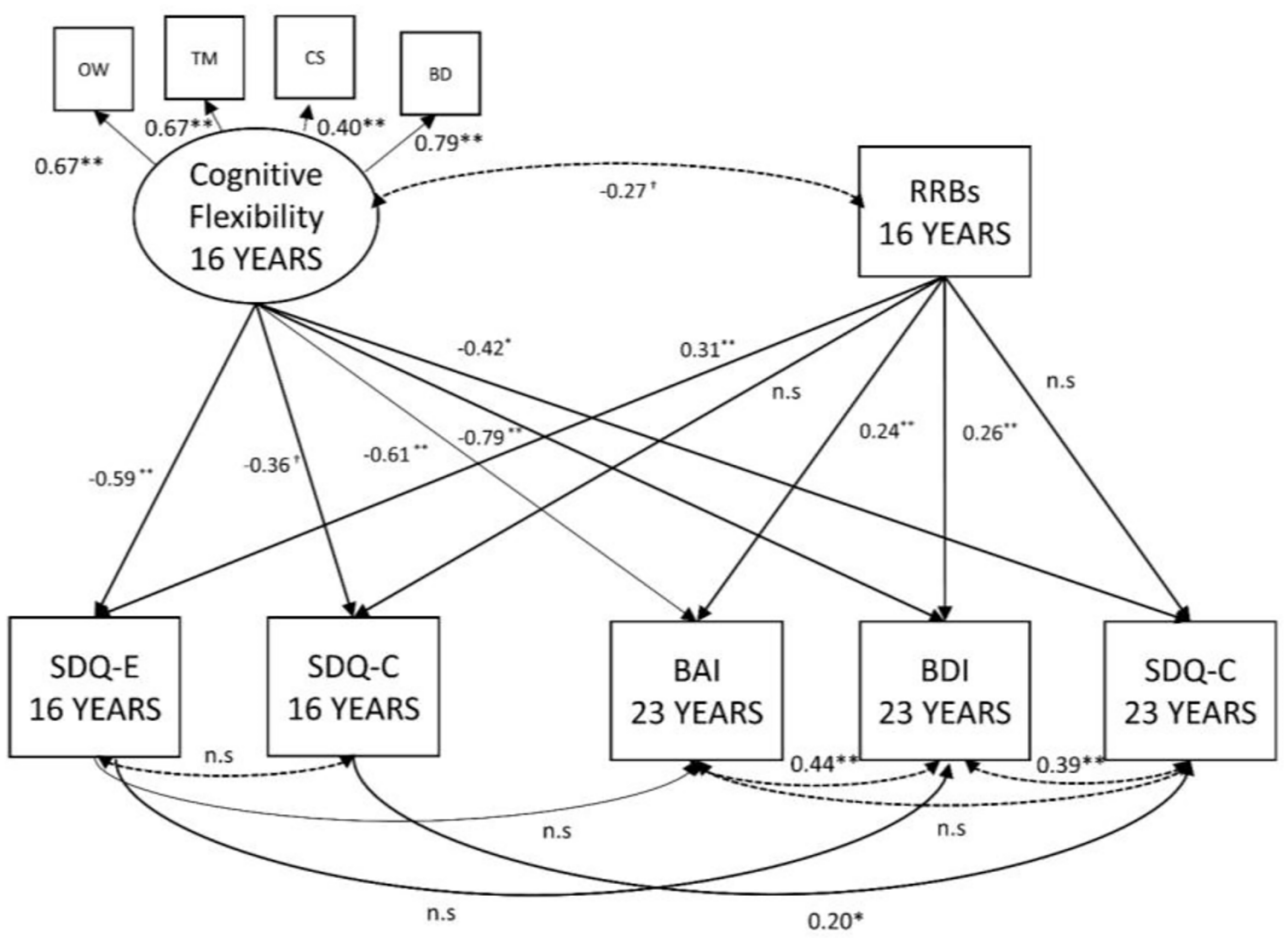
The relationship between cognitive flexibility, RRBs and emotional and behavioural symptoms at 16 and 23 years. χ^2^ (*df*) = 23.9 (26), *p* = 0.57; CFI = 1.00, RMSEA 0.00 (95% CI = 0.0–0.08). Solid lines are regression paths; dotted lines are correlations. Verbal IQ (not shown) regressed onto all variables as a covariate. OW: opposite worlds; TM: trail making; CS: card sort; BD: block design; RRBs: Social Responsiveness Scale – Restricted and Repetitive Behaviours; SDQ-E: Strengths and Difficulties Questionnaire – Emotional problems; SDQ-C: Strengths and Difficulties Questionnaire – Conduct problems; BAI: Beck Anxiety Inventory; BDI: Beck Depression Inventory. ^†^*p* ⩽ .10, *p ⩽ .05, **p ⩽ .01.

The analysis investigated the relationship between neuropsychological measures of cognitive flexibility and emotional and behavioural difficulties at both 16 and 23 years when accounting for severity of RRBs and controlling for the known association between verbal ability and both our independent and dependant variables. To effectively combine multiple inter-correlated neuropsychological measures, a ‘cognitive flexibility’ latent variable was constructed to be regressed onto each of the symptom scores in the context of a SEM. A SEM is an extension of the standard general linear model which allows the simultaneous estimation of multiple associations between independent, dependent, and latent variables. This allows the estimation of the relationship between cognitive flexibility and each mental health symptom scores across time when accounting for covariance with other predictors (i.e. RRB). The inclusion of a latent variable effectively enables us to examine the shared variance of neuropsychological tests selected based on their ability to measure elements of flexibility, thereby reducing the impact of broader executive/cognitive processes on our findings. The model included regression paths between both cognitive flexibility and RRBs and emotional and behavioural symptoms at 16 and 23 years. Within-domain paths representing the longitudinal relationship between emotional and behavioural symptoms were included so that the relationships between predictors and outcomes at 23 years accounting for symptom severity at 16 years (see Figure 1 for full model). Verbal IQ was included as a covariate and regressed onto each variable in the model.

The models were fitted to raw data using full information maximum-likelihood to account for missing data, and model fit was evaluated using the comparative fit index (CFI) and root mean square error of approximation (RMSEA). A good model fit is indicated by the above fit statistics including a chi-square likelihood ratio test *p* value ⩾ 0.05, CFI ⩾ 0.95, and an RMSEA ⩽ 0.08 (Hu & Bentler, 1999). SEM was performed in the statistical modelling software MPLUS, version 8. It is recommended that SEM analyses include approximately 10 participants for each observed variable included in the model (Bentler & Chou, 1987), but factors such as including latent variables may reduce sample size requirements (Wolf et al., 2013). Therefore, the current sample size is considered adequate for the analyses undertaken.

## Results

### Descriptive statistics

The final sample included 81 autistic young people with a mean age at Wave 2 of 15.4 years (range: 14.7–16.8 years) and an FSIQ of 83.5 (range: 50–119). The mean age of the sample at follow-up was 23.2 years (range: 21.3–25.1 years); full descriptive statistics for the sample are presented in Table 1.

### Confirmatory factor analysis of cognitive flexibility latent variable

To ensure the validity of the cognitive flexibility latent variable, a confirmatory factor analysis (CFA) was conducted. The block design (β = 0.73; *p* < .01), trail making (β = 0.73; *p* < .01) and opposite world (β = 0.72; *p* < .01) tasks all loaded strongly onto the cognitive flexibility latent variable. The card-sort task loaded significantly but to a lesser degree than the other tasks (β = 0.39; *p* < .01) but was considered adequate for inclusion in the latent variable. The weaker loading is likely explained by this variable having a binary distribution (pass/fail). Overall, the cognitive flexibility latent variable showed a good fit to the data (χ^2^ (2) = 1.6, *p* *=* 0.44; CFI = 1.00, RMSEA = 0.00).

### The relationship between neuropsychological measures of cognitive flexibility and emotional and behavioural difficulties in adolescence and early adulthood

The hypothesised model with cognitive flexibility predicting emotional and behavioural problems at ages 16 and 23 years, while accounting for RRBs at 16 years and controlling for verbal IQ, had good model fit (χ^2^ (26) = 23.9, *p* *=* 0.57; CFI = 1.0, RMSEA = 0.00; 95% CI = 0.0–0.08). Cognitive flexibility and RRBs were allowed to correlate within the model to account for their covariance (β = –0.27; *p* = .06). This model showed that at 16 years, there was a significant association between reduced cognitive flexibility and increased emotional problems (β = –0.59; *p* < .01); see Figure 1. The relationship between cognitive flexibility and behaviour problems did not reach statistical significance (β = –0.36; *p* = .06), but there was nonetheless a moderate association. The association between RRBs and emotional problems was significant (β = 0.31; *p* < .01), but the relationship with behaviour problems was not (β = 0.01; *p* = .28).

At 23 years, reduced cognitive flexibility at 16 years predicted greater behavioural problems (β = –0.42; *p* = .03), anxiety (β = –0.61; *p* < .01) and depression (β = –0.79; *p* < .01). Consistent with the cross-sectional findings, RRBs at 16 years were significantly associated with anxiety (β = 0.24; *p* = .04) and depression (β = 0.26; *p* = .03), but not behavioural problems at 23 years (β = 0.03; *p* = .91). At 23 years, there were strong inter-correlations between symptoms measures, with anxiety significantly correlated with depression (β = 0.45; *p* < .01), but not behaviour problems (β = –0.13; *p* = .33). Symptoms of depression were positively and significantly correlated with behaviour symptoms (β = 0.39; *p* < .01).

Greater verbal IQ, which was regressed onto each variable in the model as a covariate, and was found to be significantly associated with better cognitive flexibility (β = 0.69; *p* < .01) and less RRBs (β = –0.37; *p* < .01). At 16 years, verbal IQ was significantly associated with emotional (β = 0.52; *p* < .01) but not behavioural problems (β = 0.24; *p* = .16). At 23 years, verbal IQ was significantly associated with depression (β = 0.72; *p* < .01) but not anxiety (β = 0.33; *p* = .06) and behavioural problems (β = 0.01; *p* = .81).

Given the covariance between cognitive flexibility and RRBs, the model was repeated constraining the paths between RRBs and both cognitive flexibility and mental health symptoms to ‘zero’ (removing the effects of RRBs from the model). This model had relatively poor fit to the data (χ^2^ (32) = 47.0, *p* = 0.04; CFI = 0.94, RMSEA = 0.08; 95% C.I = 0.2–0.12) and had minimal impact on the relationships presented above and in Figure 1. The exception was a general increase in the standardised beta-coefficient across significant paths between cognitive flexibility and emotional and behavioural problems, and as a result, the association with behavioural problems at 16 years became significant (β = –0.41; *p* = .03).

### The role of cognitive inflexibility as a mediator of the relationship between emotional symptoms at 16 and 23 years

**Figure 2.**
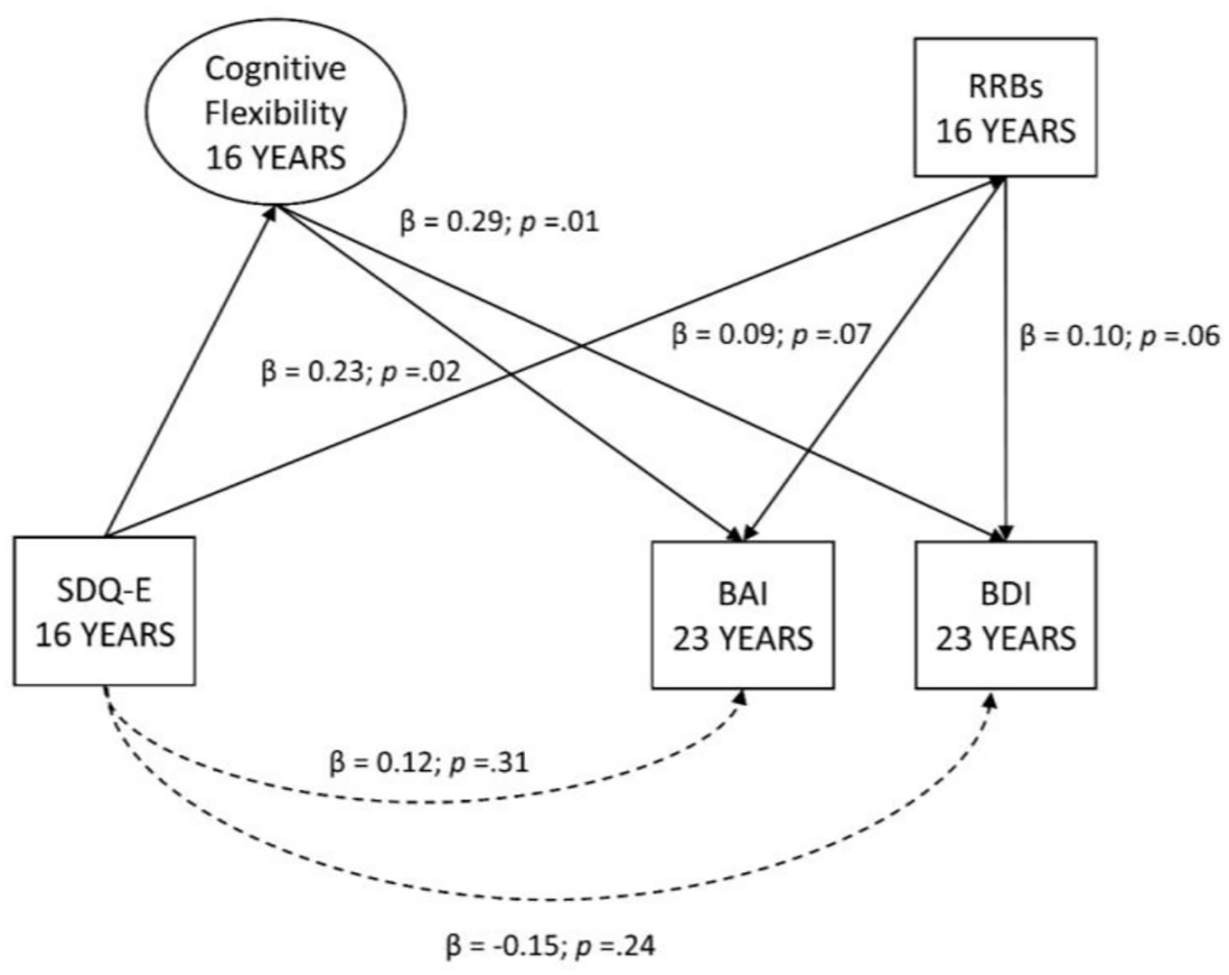
Cognitive flexibility as a mediator of the persistence of emotional symptoms.

Despite the moderate correlations (uncorrected) between emotional symptoms between ages 16 and 23 years (see Table 2), symptoms at 16 years did not predict those at 23 years in the model when cognitive flexibility and RRBs were included as predictors (SDQ-E at 16 years with BAI, β = 0.11; *p* = .38 and BAI, β = –0.16; *p* = .19 at 23 years, see Figure 1). This suggests that the stability of the relationship between emotional symptoms at 16 and 23 years may be associated with cognitive inflexibility and/or RRBs. To test this, a series of exploratory post hoc mediation analyses were conducted to examine the indirect effects (mediation) via cognitive flexibility and RRBs of emotional symptoms at 16 on anxiety and depression symptoms at 23 years (see Figure 2). The indirect path between emotional symptoms at 16 years and anxiety at 23 years, via cognitive flexibility was significant (β = 0.23; *p* = .02). The equivalent indirect path via RRBs did not reach significance (β = 0.09; *p* = .07). The same pattern was found with the indirect path between emotional symptoms at 16 years and depression at 23 years, via cognitive flexibility (β = 0.29; *p* = .01) being significant, and via RRBs not reaching significance (β = 0.10; *p* = .06).

## Discussion

The results of this study suggest a complex pattern of associations in which cognitive inflexibility is associated with emotional and behavioural difficulties across adolescence and early adulthood after accounting for their respective relationships with RRBs and verbal IQ. The finding that cognitive inflexibility was significantly associated with emotional difficulties in adolescence is consistent with much of the previous work using informant-reported behavioural rating scales (Lawson et al., 2015). This includes the wider literature showing significant associations between executive functioning, more broadly defined, anxiety (Hollocks et al., 2014) and depression (Wallace et al., 2016). However, unlike the recent study by Ozsivadjian et al. (2021), which used a parent-reported questionnaire measure of cognitive flexibility, we found a direct relationship with emotional difficulties. One explanation for this is our analysis did not include potential intermediary behavioural constructs such as intolerance of uncertainty which have been shown to have a strong relationship with anxiety in autism (Maisel et al., 2016). If this variable had been included in our model, then this may have accounted for some of the variance currently accounted for by cognitive flexibility and RRBs. Further research is required to fully understand the associations between cognitive inflexibility, intolerance of uncertainty and other constructs (i.e. alexithymia and emotional regulation) thought to be associated with emotional and behavioural difficulties, using both objective and informant-based measures.

Another inconsistency between the results presented in this article and those of Ozsivadjian et al. was the lack of a significant association between cognitive inflexibility and behavioural problems in adolescents in this study. It is possible that behavioural ratings of cognitive flexibility like those used in previous studies may be inflated by reporting bias when a young person presents with high levels of behavioural difficulties, while the objective measurement provided by neuropsychological tests is not. Furthermore, the study by Ozsivadjian et al. was based on a clinical sample, presenting to a specialist clinic, and in line with this the severity of behavioural problems as reported on the same SDQ conduct problem sub-scale was much higher than in this sample (mean score of 4.5 compared to 2.6 in this study). However, it should be noted that the association between cognitive inflexibility and behavioural difficulties was at trend level (*p* = 0.06) and significant when the confounding effect of RRBs was not accounted for in the analysis. One previous study which used both a neuropsychological and behavioural rating scale of cognitive inflexibility, found no significant association with behavioural difficulties based on the neuropsychological measure despite there being one on the behavioural rating scale (Visser et al., 2015).

A clearer pattern of association was found between cognitive inflexibility and greater scores on each of our independent symptom measures of anxiety, depression and behavioural difficulties at 23 years. This provides evidence of the importance of cognitive inflexibility in the expression of co-occurring mental health difficulties across multiple time points but also as a potential trans-diagnostic vulnerability factor across different domains of comorbidity in autism. The most consistent finding was the association between cognitive inflexibility and symptoms of anxiety. Our exploratory mediation analysis additionally suggests that cognitive inflexibility is associated with the stability of anxiety and depression from adolescence to early adulthood. As cognitive inflexibility was only measured at one timepoint, the directionality of the association with anxiety is unclear and further research is required to understand if a bi-directional association may be present. Given the relatively small sample size of this study, and its exploratory nature, this should be interpreted with caution. However, it does provide preliminary evidence to suggest that improving flexibility in adolescence may both be of benefit to concurrent symptoms and improve long-term outcomes.

A key goal of this study was to understand whether the effect of cognitive inflexibility on mental health symptoms could be separated from that of the overlapping construct of RRBs. Indeed, our findings show that RRBs were significantly associated with emotional symptoms in the same pattern as the more narrowly defined cognitive inflexibility points to an overlapping relationship, and is consistent with previous work on RRBs (Joyce et al., 2017; Rodgers et al., 2012). However, across analyses when accounting for the covariance with RRBs, the association between cognitive inflexibility and emotional symptoms both remained significant and was of a greater magnitude than that of RRBs. Therefore, while an inflexible or rigid thinking style is often encapsulated within the broader domain of RRBs, these results indicate a distinct effect. This is a first step towards a greater specificity in our understanding of factors underlying co-occurring mental health difficulties in autistic people which can be used to guide assessment and treatment by being aware of and accounting for these differences in thinking style and their impact on mental health.

### Study strengths and limitations

This study has several key strengths including a well-characterised, population-derived sample of autistic young people with confirmed diagnoses. In addition, we were able to take advantage of the fact that the sample has been studied over multiple timepoints with detailed measurement of mental health comorbidities. A further strength is the inclusion of young people with a wider range of intellectual functioning and the ability to account for this variance in our analyses.

Despite substantial strengths, there are several study limitations that require consideration when interpreting the current findings. First, while the focus of this analysis was on the use of neuropsychological measures of cognitive inflexibility, in order to fully understand its measurement and relationships with informant-based behavioural reports, both types of measurement are required in the same sample. This was not possible in the current sample, but future studies should focus on combining measurement of cognitive inflexibility from different sources to investigate consistency in their relationships with symptoms of emotional and behavioural difficulties. It is also important to consider that while our neuropsychological measures were selected to all include a component of flexibility (i.e. set-shifting and attentional control/flexibility), the nature of these tasks means they also measure other elements of executive functioning. This highlights the need for studies to further refine the construct of cognitive inflexibility using measurement across different levels and across informants.

Second, while the use of a range of well-validated measures of mental health symptoms is a strength, the measures available at age 16 were limited and meant we could not study the association with cognitive inflexibility with more specificity. For example, it would have been beneficial to have independent measures of both anxiety and depression rather than relying on the SDQ emotional problems sub-scale which collapses both sets of symptoms together. Similarly, we were limited to using the SDQ conduct problems sub-scale across both timepoints. Future research would benefit from using a more detailed set of measures to be able to tease apart whether the relationship with cognitive inflexibility is related to more overt conduct behaviours (i.e. overt oppositional or aggressive behaviours) or emotional dysregulation/irritability. Similarly, our use of the SRS as a measure of RRBs may also be considered a limitation. Future research would benefit from a more comprehensive assessment of RRBs to ensure that their association with mental health has not been underestimated. For instance, alternative measures such as the Repetitive Behaviours Scale – Revised (RBS-R; Lam & Aman, 2007) or detailed observation may provide a more comprehensive assessment of RRBs.

Finally, despite the fact the sample is well characterised, and the sample size adequate, for the current analyses undertaken, the overall sample size may be considered small. This also poses a challenge for understanding sub-groups within the sample. For example, we did not have adequate power to stratify the sample based on level of intellectual disability. While having a sample which represents the wide ability range of autistic people can be considered an advantage, further research is needed to understand how the mechanisms underlying emotional and behavioural difficulties may differ in these groups. The lack of comparison group means that our results are limited to understanding the role of cognitive inflexibility in autistic people. Future research should investigate whether cognitive inflexibility plays a similar role in neurotypical individuals. Therefore, the results described above would benefit from replication in a larger, and independent, data set with a suitable comparison group.

### Clinical implications

The identification of cognitive inflexibility as an important mechanism in both emotional and behavioural comorbidities in autistic people is an important step forward in developing personalised or adapted intervention approaches. For some individuals, an inflexible thinking style may reduce the benefits of interventions currently thought to be effective such as adapted cognitive-behavioural therapy (Wood et al., 2020). Importantly, the evidence to date suggest that cognitive inflexibility may be a trans-diagnostic cognitive process which may help explain why autistic people are at increased risk of a range of co-occurring mental health difficulties. In those without, autism inflexible thinking has been shown to be related to a range of difficulties including anxiety, depression, substance use, and eating disorders, as well as the presence of multiple co-occurring conditions (Levin et al., 2014; Tchanturia et al., 2011). The association between cognitive inflexibility and mental health problems may suggest that either specifically targeting this construct through intervention or promoting flexibility through the process of cognitive or behavioural therapies may be of benefit. The possibility that cognitive inflexibility may have a longitudinal impact on mental health suggests that future research to develop intervention approaches that build resilience by increasing flexibility may be indicated.

There is already some evidence that a group-based educational intervention targeting executive functioning can lead to improvements in cognitive flexibility in autistic young people (Kenworthy et al., 2014). It is possible that this, or other interventions focusing of cognitive mechanisms such as cognitive remediation therapy (Dandil et al., 2020), could be further adapted to treat mental health comorbidities in autism. This could be used as an adjunct therapy to standard cognitive behavioural therapy (CBT) approaches or as a stand-alone treatment for those who do not engage well with more traditional therapeutic approaches. If, as suggested above, future research can identify a well-validated set of measures of cognitive inflexibility, it may be possible to stratify people into those who may benefit from an intervention focused on increasing flexibility.

## Conclusion

In conclusion, cognitive inflexibility when assessed using neuropsychological measures is associated with increased emotional and behavioural difficulties in autism. The association between cognitive inflexibility and the severity of these symptoms may vary across time and are to some degree independent of the severity of RRBs and verbal IQ. This shows promise for the development of personalised approaches to the treatment of co-occurring mental health difficulties for autistic people by targeting specific cognitive mechanisms.
